# Effects of Rosuvastatin on Apolipoprotein J in Balloon-Injured Carotid Artery in Rats

**DOI:** 10.5935/abc.20180163

**Published:** 2018-10

**Authors:** Ning Yang, Bo Dong, Jinyu Yang, Yang Li, Lu Kou, Yue Liu, Qin Qin

**Affiliations:** Department of Cardiovascular, Tianjin Chest Hospital, Tianjin - China

**Keywords:** Coronary Artery Dsease, Percutaneous Coronary Intervention, Rosuvastatin Calcium, Apolipoprotein J, Coronary Reestenosis, Rats

## Abstract

**Background:**

Restenosis after percutaneous coronary intervention in coronary heart disease remains an unsolved problem. Clusterin (CLU) (or Apolipoprotein [Apo] J) levels have been reported to be elevated during the progression of postangioplasty restenosis and atherosclerosis. However, its role in neointimal hyperplasia is still controversial.

**Objective:**

To elucidate the role Apo J in neointimal hyperplasia in a rat carotid artery model *in vivo* with or without rosuvastatin administration.

**Methods:**

Male Wistar rats were randomly divided into three groups: the control group (n = 20), the model group (n = 20) and the statin intervention group (n = 32). The rats in the intervention group were given 10mg /kg dose of rosuvastatin. A 2F Fogarty catheter was introduced to induce vascular injury. Neointima formation was analyzed 1, 2, 3 and 4 weeks after balloon injury. The level of Apo J was measured by real-time PCR, immunohistochemistry and western blotting.

**Results:**

Intimal/medial area ratio (intimal/medial, I/M) was increased after balloon-injury and reached the maximum value at 4weeks in the model group; I/M was slightly increased at 2 weeks and stopped increasing after rosuvastatin administration. The mRNA and protein levels of Apo J in carotid arteries were significantly upregulated after rosuvastatin administration as compared with the model group, and reached maximum values at 2 weeks, which was earlier than in the model group (3 weeks).

**Conclusion:**

Apo J served as an acute phase reactant after balloon injury in rat carotid arteries. Rosuvastatin may reduce the neointima formation through up-regulation of Apo J. Our results suggest that Apo J exerts a protective role in the restenosis after balloon-injury in rats.

## Introduction

Coronary heart disease (CHD) is one of the most common cardiovascular diseases with high morbidity and mortality. Major effective techniques for myocardial revascularization are percutaneous coronary intervention (PCI) and coronary bypass surgery. Percutaneous transluminal coronary angioplasty (PTCA) is an effective treatment for CHD, but its effect in long-term is influenced by a high restenosis rate. Although drug eluting stents (DES) combined with dual antiplatelet therapy greatly reduce the occurrence of restenosis, the incidence rate still exceeds 10%.^1^^,^^2^ The mechanism underlying restenosis after PCI has been widely studied worldwide, but effective cellular or molecular targets for the treatment of restenosis after PCI urgently needs to be identified.

Clusterin (CLU), or Apolipoprotein (Apo) J, is a heterodimeric glycoprotein, which is composed of α and β subunits linked by disulfide bond.^3^^,^^4^ The coding gene of Apo J is located on chromosome 8p21-p12, mainly encoding two isoforms - secretory CLU (sCLU) and nuclear CLU (nCLU).^5^ Apo J has been reported to be induced during the progression of postangioplasty restenosis and atherosclerosis.^6^^-^^9^ However, the role of Apo J in neointimal hyperplasia is still controversial. It has been reported that Apo J could stimulate the proliferation and migration of vascular smooth muscle cell (VSMC) in CLU-knockout mice by inhibiting the expression of p53 and p21, and promote restenosis.^10^^,^^11^ On the contrary, Kim et al.^12^ revealed that the overexpression of sCLU can inhibit the migration and proliferation of VSMC and inhibit the apoptosis of cells. In view of existing paradoxical findings, we aimed to elucidate the role Apo J in neointimal hyperplasia in a rat carotid artery model *in vivo* with or without rosuvastatin intervention.

## Methods

### Animals

Male Wistar rats weighing 350-400 g were randomly divided into three groups: control group (n = 20), model group (n = 20) and intervention (statin) group (n = 32). All animals were then randomly divided into 4 groups - to be evaluated at 1, 2, 3 or 4 weeks after balloon injury. The study was approved by the Ethics Committee of Tianjin Chest Hospital.

### Balloon injury

The rats were weighed on the day of operation, and randomly divided into three groups. The rats in the intervention group were given 10 mg/kg dose of rosuvastatin. A 2F Fogarty catheter was introduced to induce vascular injury as previously reported.^13^ Briefly, the rats were anesthetized after intraperitoneal injection of 10% chloral hydrate at a dose of 0.3 mL/100 g body weight. A 2F balloon catheter was inserted into aortic outlet of carotid artery. The balloon was then inflated and pulled back 3 times to denude the endothelium.

At 1, 2, 3 and 4 weeks after surgery, rats were anesthetized by intraperitoneal injection of 10% chloral hydrate at a dose of 0.3 ml/100 g body weight. Then, the animals were sacrificed by intravenous administration of 2-3 mL of potassium chloride solution *via* subclavian vein; 0.3 cm of the right carotid artery was fixed in 10% neutral formalin for pathological examination, and the other part was frozen immediately in liquid nitrogen and stored at -80ºC for further use.

### Hematoxylin-eosin (HE) staining

Vascular specimens were fixed in 10% formaldehyde solution for 3-4h. Routine dehydration and paraffin embedding were performed. The sections were cut evenly and the thickness was 4 µm. The injury of blood vessels was observed under light microscope.

### Immunohistochemistry (IHC) assay

The level of Apo J was assessed by IHC in rat carotid artery. The primary antibody (polyclonal rabbit anti-human Apo J IgG) was purchased from Santa Cruz, Inc. (Cat No. sc-8354). The secondary antibody (labeled goat anti-rat/rabbit IgG polymer) was purchased from Maixin BioTech (Fuzhou, China). All photos were captured and saved using the ISCapture system, and data collection and analysis are performed using the Image Pro Plus 6 image processing software.

### Enzyme-linked immunosorbent assay (ELISA)

Venous blood was collected and centrifugated at 3000r/min for 10 min. The supernatant was collected using a micropipette and stored in the refrigerator at -20ºC for use. The samples were then thawed at room temperature for ELISA. ELISA was performed using a commercial kit (Rat Competitive ELISA for Apolipoprotein J A 252 SC), following the manufacturers’ instructions.

### Real-time polymerase chain reaction (PCR)

The mRNA level of Apo J was detected by real-time PCR in rat carotid artery. RNA was extracted by Trizol one-step extraction method, and reverse transcription was performed. Primers used for amplification for Apo J were as follows: Forward, TAA GGA GAT TCA GAA CGC CG; reverse, ATC CCT GGT GTC ATC TAG AG. Primers for the control GAPDH were as follows: Forward, GTG ATG CTG GTG CCG AGT AG; reverse, GGT GGC AGT GAT GGC GTG C. Real-time PCR reactions were prepared following the instructions of SYBR^®^ *Premix Ex Taq*™ system (Perfect Real Time). The mRNA levels in each sample were calculated by 2^-ΔΔCt^.

### Western blotting

Proteins were extracted from 30 mg of rat carotid artery. Briefly, proteins were separated using SDS-PAGE with 10% separation gel and 5% concentrated gel. Then the separated proteins were transferred into polyvinylidene difluoride (PVDF) membranes. The membranes were blocked and incubated with antibodies. Relative levels of Apo J were analyzed using Image Lab analysis software. β-actin was used as inner control. Bands were quantified using QUANTITY ONE software (Bio-Rad, Hercules, CA, USA).

### Statistical analysis

Statistical analysis was performed using SPSS 20.0. Quantitative data were expressed by mean ± standard deviation (SD). The difference between two groups was compared using independent-samples *t* test; comparisons between three groups were analyzed using one-way ANOVA (analysis of variance). P *<* 0.05 was considered statistically significant.

## Results

### Survival and success rates of rat carotid artery model

Among the 52 rats of the model group and the intervention group, 2 died during operation by suffocation, and 2 died for arterial hemorrhage 12h after operation. Therefore, 47 rats survived with approximately 90% survival rate. The pathological examination showed intimal hyperplasia and thickening in the experimental group, suggesting that the model was successfully constructed. The mean operation time was 34.19 ± 6.09 min. The feasibility and success rate of this model can be highly reproducible if surgical procedures are properly performed.

### Level of serum Apo J

There was no significant difference in serum Apo J level before and after operation in the intervention group (Table 1). There was no significant difference in the level of serum Apo J at 1, 2, 3 and 4 weeks before (*F*= 1.002, p = 0.408) of after (*F*= 0.189, p = 0.903) operation.

**Table 1:** Serum levels of apolipoprotein J (Apo J) before and after operation in the statin intervention group

Time points	Pre-operation		Post-operation		t^*^	p^#^
	n	Apo J	n	Apo J		
1	7	13.498 ± 3.015	7	10.317 ± 3.567	1.802	0.097
2	7	14.062 ± 4.538	7	11.516 ± 1.762	1.383	0.192
3	8	11.234 ± 2.740	8	11.117 ± 3.104	0.08	0.937
4	8	14.143 ± 4.609	8	11.205 ± 3.579	1.424	0.176
F^$^		1.002		0.189		
*P**^#^***		0.408		0.903		

^*^ t test used to compare the differences between the two groups;

^$^ F one-way ANOVA (analysis of variance) to compare the difference between all four groups.

^#^ p value < 0.05 was considered statistically significant.

### Statin intervention inhibited intimal hyperplasia

**Figure 1:**
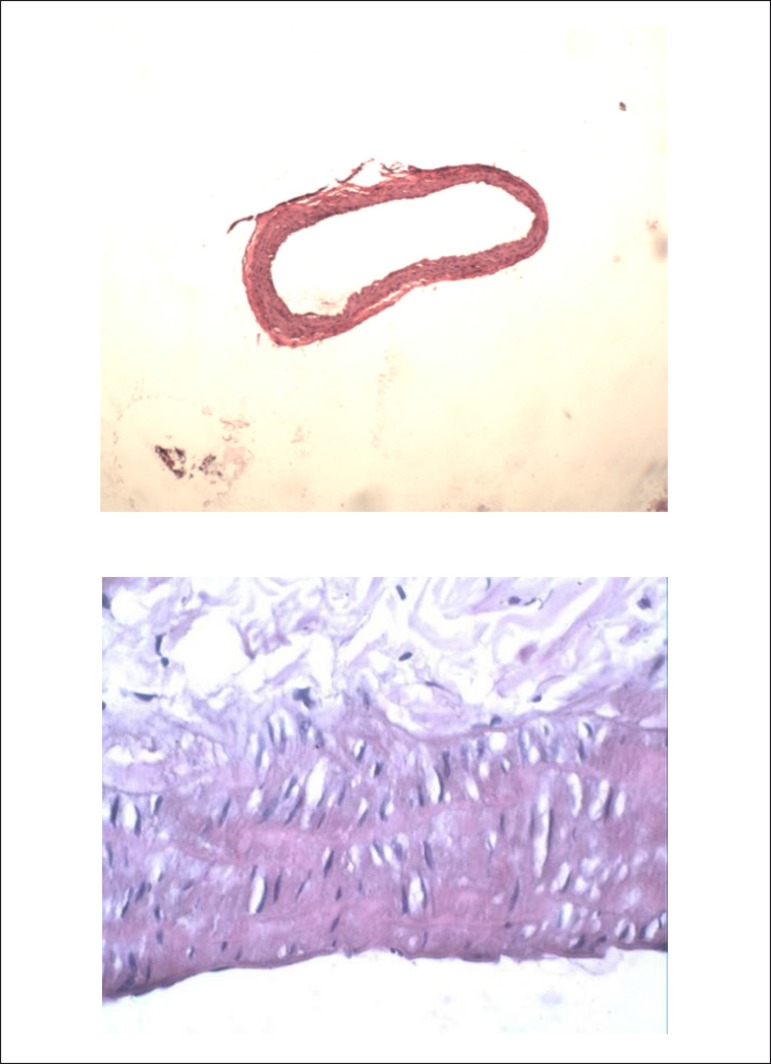
Hematoxylin-eosin (HE) staining in the control group. Upper, magnification 40×; lower, magnification 400×.

**Figure 2:**
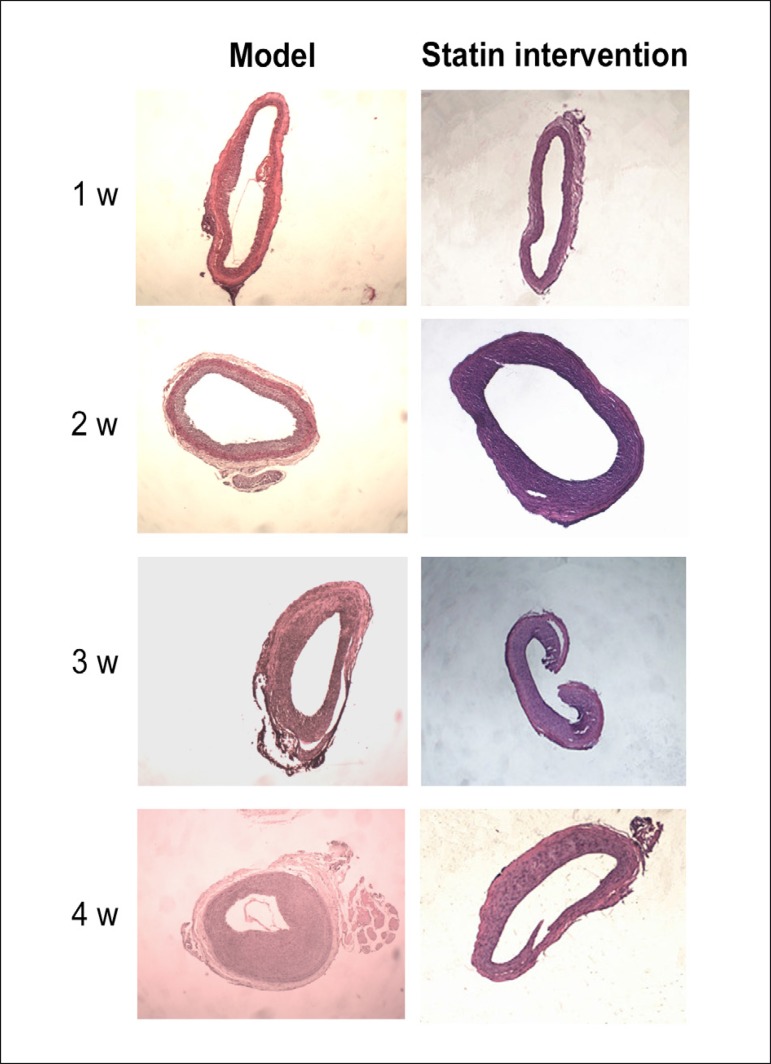
Hematoxylin-eosin (HE) staining in the model group and in intervention group 1 week (w), 2 weeks, 3 weeks and 4 weeks after balloon injury of rat carotid arteries; magnification 40×.

**Figure 3:**
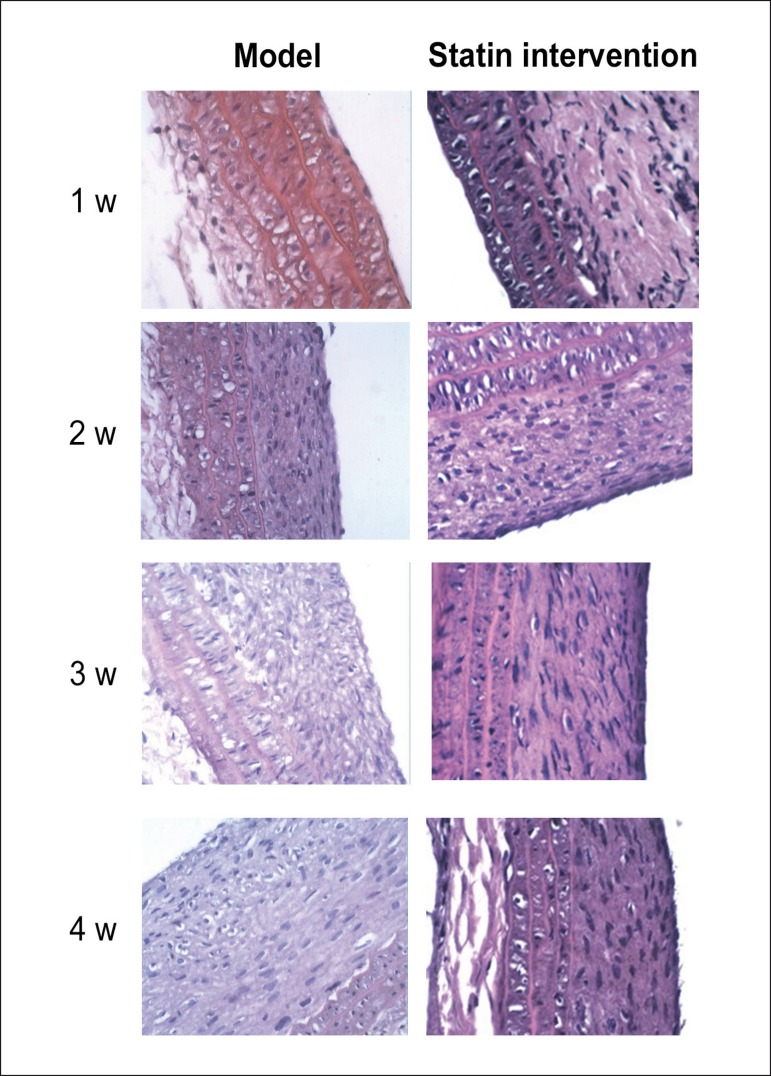
Hematoxylin-eosin (HE) staining in the model group and in intervention group 1 week (w), 2 weeks, 3 weeks and 4 weeks after balloon injury of rat carotid arteries; magnification 400×.

Results of pathological examination showed that no intimal hyperplasia was observed in the control group (Figure 1). In the model group and intervention group, the intima was slightly thickened 1 week after operation, and further thickened 2 weeks after operation. During 3 weeks after operation, the degree of intimal hyperplasia was further aggravated in the model group; however, this change was not as marked as at 2 weeks after operation, and the cells gradually became paralleled. During 4 weeks after operation, the degree of intimal hyperplasia was further aggravated in the model group, but no significant changes were observed regarding the degree of intimal hyperplasia as compared with week 3 (Figure 2 and 3).

Intimal and medial membrane areas were measured using Image Pro Plus 6, and intimal/medial area ratio (intimal/medial, I/M) was used to indicate the degree of intimal hyperplasia. As shown in Table 2, the I/M was close to 0 in the control group and was significantly different from that in the model group and the intervention group at all time points (1, 2, 3 and 4 weeks). There were significant differences of I/M between different time points in the model group, and I/M reached the maximum at the fourth week. No significant difference of I/M was observed between 2, 3 and 4 weeks post-surgery in the intervention group, and I/M in the intervention group was significantly lower than that in the model group (Table 2). Taken together, our results suggest that rosuvastatin could significantly inhibit intimal hyperplasia in rats.

**Table 2:** Intimal/medial (I/M) area ratio in the study groups

Time points	Control group		Model group		Statin intervention group		t^*^	p*^#^*
	n	I/M	n	I/M	n	I/M		
1	5	0.04 ± 0.07	5	0.63 ± 0.40^γ^	5	0.42 ± 0.04^γ^	10.066	< 0.001
2	5	0.01 ± 0.02	4	1.08 ± 0.29^▲^	4	1.29 ± 0.31^∆▲^	39.639	< 0.001
3	5	0.03 ± 0.03	4	1.81 ± 0.11^aβ^	4	1.47 ± 0.54^∆β^	37.142	< 0.001
4	5	0.05 ± 0.04	4	2.61 ± 1.12^abθ^	4	1.50 ± 0.26^∆θc^	20.287	< 0.001
F^$^		0.741		9.432		21.393		
*P**^#^***		0.543		< 0.001		< 0.001		

^*^ t test used to compare the differences between the two groups.

^$^ F one-way ANOVA (analysis of variance) to compare the difference between all four groups.

^#^ p value < 0.05 was considered statistically significant

### Level of Apo J in carotid arteries

**Figure 4:**
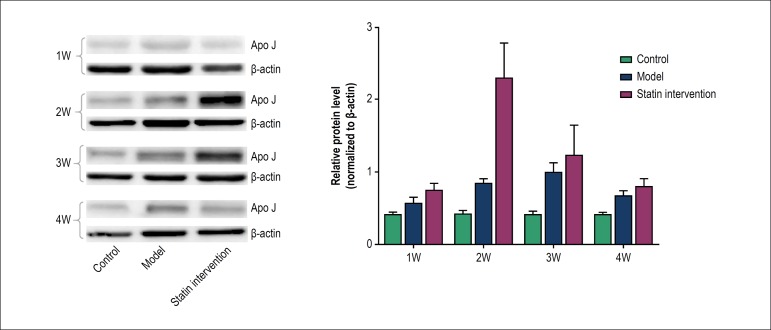
Western blotting of apolipoprotein J (Apo J) protein levels 1 week (w), 2 weeks, 3 weeks and 4 weeks after balloon injury of rat carotid arteries.

The mRNA levels of Apo J were measured by real-time PCR. Our results showed that the Apo J mRNA level was strikingly increased 2 weeks after operation, reached to a peak at the 3^rd^ week, and decreased at the 4^th^ week post-surgery in the model group. In intervention group, the Apo J mRNA level was strikingly increased and reached to a peak at the 2^nd^ week, and decreased at the 3^rd^ and 4^th^ week post-surgery in the intervention group. In addition, the mRNA level of Apo J was higher in the intervention group than in the model group at the 1^st^ week after operation. At the 2^nd^ week post-surgery, the mRNA level of Apo J was strikingly increased in both groups and was significantly higher in the stain-intervention group than the model group (Table 3). Similar results have been observed in the protein levels of Apo J as shown in Figure 4. Our results showed that rosuvastatin could significantly increase the expression level of Apo J in balloon-injured rat carotid arteries.

**Table 3:** Relative (2-ΔΔCt) levels of apolipoprotein J mRNA

Time points	Control group		Model group		Statin intervention group		t^*^	p*^#^*
	n	2-ΔΔCt	n	2-ΔΔCt	n	2-ΔΔCt		
1	5	0.958 ± 0.251	5	0.641 ± 0.296	6	1.275 ± 0.468^a^	4.212	0.039
2	5	0.948 ± 0.090	4	7.804 ± 1.328^a▲^	6	10.040 ± 2.086^∆b^	52.279	< 0.001
3	5	1.004 ± 0.196	4	8.011 ± 2.306^aβ^	6	7.327 ± 2.869^∆^^*^^β^	15.31	< 0.001
4	5	1.048 ± 0.349	4	3.429 ± 1.119^abcθ^	6	2.413 ± 0.492^*^^#^^θ^	14.212	0.001
F^$^		0.182		29.266		31.336		
*P**^#^***		0.907		< 0.001		< 0.001		

^*^ t value was calculated using independent-samples t test to compare the difference between two groups.

^$^ F value was calculated using one-way ANOVA (analysis of variance) to compare the difference among the four groups.

^#^ p value (probability value) < 0.05 is considered to be statistically significant.

## Discussion

In the present study, we found that I/M increased after balloon-injury and reached the maximum at 4w in the model group; also, I/M was slightly increased at 2w and stopped increasing after rosuvastatin administration. Our results suggest that rosuvastatin could significantly reduce the degree of intimal hyperplasia in balloon-injured carotid arteries in rats. The levels of Apo J mRNA and protein in carotid arteries were significantly upregulated after rosuvastatin administration as compared with the model group, and reached to maximum at 2 weeks, which was earlier than the in the model group. Our results suggest that rosuvastatin may inhibit intimal hyperplasia through upregulation of Apo J after balloon-injury in rats.

Apo J has been reported to be closely related to cardiovascular diseases, such as atherosclerosis and restenosis after angioplasty.^14^^,^^15^ Ishikawa et al.^7^ revealed the distribution of Apo J in the extracellular matrix of endarterium in human atherosclerotic aorta, and its potential protective role against human atherosclerosis by cholesterol transport from the aortic wall to the liver. It has been reported that ApoJ is increased in tissue injury and cell stress, and plays vital role in protection against oxidative stress, cell lysis and apoptotic cell death.^16^^-^^21^ Additionally, ApoJ could be observed in active tissue remodeling. These findings indicate that ApoJ may act as an acute phase reactant. In the present study, we observed a marked neointimal thickening 2 weeks post-surgery, with proliferation and migration VSMCs observed by HE staining in the model group. The proliferation and migration of VSMC were the most active at week 3, and decrease at week 4. In the meantime, the mRNA and protein levels of Apo J were significantly increased at week 2, reached a peak at 3 weeks after operation, and then decreased at 4 weeks. The results showed high expression of Apo J in the phase of active proliferation and migration of VSMCs. Consistent with other studies, our results suggest that Apo J may be an acute phase reactant after balloon-injury in rat carotid arteries.

In-stent restenosis after interventional procedures has become one of the most urgent problems to be solved worldwide. Rosuvastatin, a potent hydroxymethylglutaryl coenzyme A (HMG-CoA) reductase inhibitor, has been reported to reduce neointimal thickening after vascular endothelial injury in rats^13^ In the present study, the rats in the intervention group received intragastric administration of rosuvastatin (10 mg/kg/d). In accordance with other studies,^22^^-^^24^ we found that rosuvastatin significantly reduced the neointima formation.

It has been reported that secreted isoform of Apo J (sCLU) could inhibit the proliferation and migration of VSMCs.^12^^,^^25^ Kim et al.^12^ also found that Apo J could significantly inhibit neointimal hyperplasia using adenovirus-mediated overexpression of Apo J in rats. In the present study, we found that the mRNA and protein levels of ApoJ in carotid arteries were significantly upregulated after rosuvastatin administration as compared with the model group. Moreover, Apo J reached a maximum at week 2 after rosuvastatin administration, and that was earlier than the model group which reached peak expression at the third week. These results suggest that rosuvastatin may increase the level of Apo J in the balloon-injured carotid arteries, which indirectly indicates a protective role of Apo J against restenosis after balloon-injury in rats.

## Conclusion

Our results showed that Apo J served as an acute phase reactant after balloon-injury in rat carotid arteries. Rosuvastatin may reduce the neointima formation through further up-regulation of Apo J. Our findings suggest that Apo J exerts a protective role against restenosis after balloon-injury in rats.

## References

[1] Sharma PK, Chhatriwalla AK, Cohen DJ, Jang JS, Baweja P, Gosch K (2017). Predicting long-term bleeding after percutaneous coronary
intervention. Catheter Cardiovasc Interv.

[2] Lee JY, Park DW, Kim YH, Yun SC, Kim WJ, Kang SJ (2011). Incidence, predictors, treatment, and long-term prognosis of
patients with restenosis after drug-eluting stent implantation for
unprotected left main coronary artery disease. J Am Coll Cardiol.

[3] Shannan B, Seifert M, Boothman DA, Tilgen W, Reichrath J (2006). Clusterin and DNA repair: a new function in cancer for a key
player in apoptosis and cell cycle control. J Mol Histol.

[4] Trougakos IP, Gonos ES (2006). Regulation of clusterin/apolipoprotein J, a functional homologue
to the small heat shock proteins, by oxidative stress in ageing and
age-related diseases. Free Radic Res.

[5] Park S, Mathis KW, Lee IK (2014). The physiological roles of apolipoprotein J/clusterin in
metabolic and cardiovascular diseases. Rev Endocr Metab Disord.

[6] Gelissen IC, Hochgrebe T, Wilson MR, Easterbrook-Smith SB, Jessup W, Dean RT (1998). Apolipoprotein J (clusterin) induces cholesterol export from
macrophage-foam cells: a potential anti-atherogenic
function?. Biochem J.

[7] Ishikawa Y, Akasaka Y, Ishii T, Komiyama K, Masuda S, Asuwa N (1998). Distribution and synthesis of apolipoprotein J in the
atherosclerotic aorta. Arterioscler Thromb Vasc Biol.

[8] Navab M, Anantharamaiah GM, Reddy ST, Van Lenten BJ, Wagner AC, Hama S (2005). An oral apoJ peptide renders HDL antiinflammatory in mice and
monkeys and dramatically reduces atherosclerosis in apolipoprotein E-null
mice. Arterioscler Thromb Vasc Biol.

[9] Miyata M, Biro S, Kaieda H, Eto H, Orihara K, Kihara T (2001). Apolipoprotein J/clusterin is induced in vascular smooth muscle
cells after vascular injury. Circulation.

[10] Millis AJ, Luciani M, McCue HM, Rosenberg ME, Moulson CL (2001). Clusterin regulates vascular smooth muscle cell nodule formation
and migration. J Cell Physiol.

[11] Shirasawa T, Miyata M, Eto H, Hamada N, Akasaki Y, Miyauchi T (2009). Deficiency of clusterin inhibits neointimal hyperplasia after
vascular injury. J Atheroscler Thromb.

[12] Kim HJ, Yoo EK, Kim JY, Choi YK, Lee HJ, Kim JK (2009). Protective role of clusterin/apolipoprotein J against neointimal
hyperplasia via antiproliferative effect on vascular smooth muscle cells and
cytoprotective effect on endothelial cells. Arterioscler Thromb Vasc Biol.

[13] Preusch MR, Vanakaris A, Bea F, Ieronimakis N, Shimizu T, Konstandin M (2010). Rosuvastatin reduces neointima formation in a rat model of
balloon injury. Eur J Med Res.

[14] Garcia-Rodriguez S, Arias-Santiago S, Perandres-Lopez R, Orgaz-Molina J, Castellote L, Buendia-Eisman A (2013). Decreased plasma levels of clusterin in patients with
psoriasis. Actas Dermosifiliogr.

[15] Yanni AE, Agrogiannis G, Gkekas C, Perrea D (2014). Clusterin/Apolipoprotein J immunolocalization on carotid artery
is affected by TNF-alpha, cigarette smoking and anti-platelet
treatment. Lipids Health Dis.

[16] Witte DP, Aronow BJ, Stauderman ML, Stuart WD, Clay MA, Gruppo RA (1993). Platelet activation releases megakaryocyte-synthesized
apolipoprotein J, a highly abundant protein in atheromatous
lesions. Am J Pathol.

[17] Sivamurthy N, Stone DH, Logerfo FW, Quist WC (2001). Apolipoprotein J inhibits the migration, adhesion, and
proliferation of vascular smooth muscle cells. J Vasc Surg.

[18] Foglio E, Puddighinu G, Fasanaro P, D’Arcangelo D, Perrone GA, Mocini D (2015). Exosomal clusterin, identified in the pericardial fluid, improves
myocardial performance following MI through epicardial activation, enhanced
arteriogenesis and reduced apoptosis. Int J Cardiol.

[19] Van Dijk A, Vermond RA, Krijnen PA, Juffermans LJ, Hahn NE, Makker SP (2010). Intravenous clusterin administration reduces myocardial infarct
size in rats. Eur J Clin Invest.

[20] Lee YN, Shim YJ, Kang BH, Park JJ, Min BH (2012). Over-expression of human clusterin increases stress resistance
and extends lifespan in Drosophila melanogaster. Biochem Biophys Res Commun.

[21] Pereira RM, Mekary RA, da Cruz Rodrigues KC, Anaruma CP, Ropelle ER, da Silva AS (2018). Protective molecular mechanisms of clusterin against apoptosis in
cardiomyocytes. Heart Fail Rev.

[22] van der Harst P, Groenewegen HC, Roks AJ, Buikema H, Zijlstra F, van Gilst WH (2008). Rosuvastatin attenuates angiotensin II-induced neointimal
formation after stent implantation in the rat. Coron Artery Dis.

[23] Kappert K, Leppanen O, Paulsson J, Furuhashi M, Carlsson MA, Heldin CH (2006). Highly active antiretroviral therapy attenuates
re-endothelialization and alters neointima formation in the rat carotid
artery after balloon injury. J Acquir Immune Defic Syndr.

[24] Luan Z, Chase AJ, Newby AC (2003). Statins inhibit secretion of metalloproteinases-1, -2, -3, and -9
from vascular smooth muscle cells and macrophages. Arterioscler Thromb Vasc Biol.

[25] Miwa Y, Takahashi-Yanaga F, Morimoto S, Sasaguri T (2004). Involvement of clusterin in 15-deoxy-delta12,14-prostaglandin
J2-induced vascular smooth muscle cell differentiation. Biochem Biophys Res Commun.

